# Review on the Biological Degradation of Polymers in Various Environments

**DOI:** 10.3390/ma13204586

**Published:** 2020-10-15

**Authors:** Silvia Kliem, Marc Kreutzbruck, Christian Bonten

**Affiliations:** Institut für Kunststofftechnik, University of Stuttgart, Pfaffenwaldring 32, 70569 Stuttgart, Germany; marc.kreutzbruck@ikt.uni-stuttgart.de (M.K.); christian.bonten@ikt.uni-stuttgart.de (C.B.)

**Keywords:** biodegradable polymers, environmental pollution, end of life scenario, plastics in the environment

## Abstract

Biodegradable plastics can make an important contribution to the struggle against increasing environmental pollution through plastics. However, biodegradability is a material property that is influenced by many factors. This review provides an overview of the main environmental conditions in which biodegradation takes place and then presents the degradability of numerous polymers. Polylactide (PLA), which is already available on an industrial scale, and the polyhydroxyalkanoates polyhydroxybutyrate (PHB) and polyhydroxybutyrate-co-valerate (PHBV), which are among the few plastics that have been proven to degrade in seawater, will be discussed in detail, followed by a summary of the degradability of further petroleum-, cellulose-, starch-, protein- and CO_2_-based biopolymers and some naturally occurring polymers.

## 1. Introduction

Plastics have become an indispensable part of everyday life. The various strengths of plastics come into their own in a wide variety of applications—packaging, clothing, car tires and much more. At the same time, however, the often-desired resistance to environmental influences and other stresses represents a major challenge. Plastic waste is increasingly found in the oceans, in rivers and in the ground, mostly as a result of human misconduct. Animals and humans ingest plastic particles with their food; the consequences are not yet foreseeable [1]. Rethinking of society is required in order to reduce the further input of plastics into the environment [2].

In the media [3], it is repeatedly reported that polymers, and thus also plastics, would need several hundred years to be degraded in the environment. It is not yet known which studies these statements are based on, and the authors assume that these are estimates only. In addition, there is repeatedly the opinion that biopolymers, as well as plastics and fibers based on them, offer a solution to this social problem. The term biopolymers covers both biobased and biodegradable polymers. Biobased polymers are produced based on renewable raw materials, and can thus reduce the release of fossil CO_2_ emissions from crude oil. However, the biodegradability of some biopolymers, as well as plastics and synthetic fibers based on them, has nothing to do with whether their carbon source originates from fossil fuel or renewable sources [4].

Biodegradability was proven, for example, according to EN 13432 [5] and ASTM D6400 [6] by CO_2_ release. The percentage of biodegradation results from the ratio of CO_2_ produced by the test substance to the maximum theoretical amount of CO_2_ that can be produced by the test substance [7]. The evaluation of the results is based on different methods: The most common approach in literature is to examine the total mass loss. However, a decrease in mass can be based on many factors, especially a loss due to mechanical abrasion. It should, therefore, be anticipated at this point that the measurement of the released CO_2_ under defined laboratory conditions is the only reliable method to determine the actual biological degradation [8]. In order to be sure that the produced CO_2_ is derived directly from the degraded polymer, and not from the surrounding biomass, it is possible to use polymer variants with ^13^C-labeled carbon in all its monomers. This allows the polymer-derived carbon to be traced [9]. However, this method is costly and labor-intensive, and cannot be applied widely.

Numerous research projects deal with the biological degradation of various polymers under different environmental conditions. The aim of this review is to provide a comprehensive overview of studies on the biological degradation of the most important biopolymers and to prove the necessary conditions for the successful degradation of each polymer on the basis of literature data. First, the definition and the influencing factors of biological degradation will be discussed.

## 2. Biological Degradation

Biological degradation is understood as the degradation of complex organic matter into carbon dioxide, methane, water, minerals and new biomass by means of a biological metabolic process [7]. This is achieved by the enzymatic activity of certain microorganisms, in particular bacteria and fungi, which first colonize the surface of the plastic and secrete a biofilm of specific enzymes. The excreted enzymes split the long polymer chains into short chain fragments, which are transported by means of tunnel proteins in the cell wall into the interior of the microorganism where they can be metabolized [10].

The degradation behavior depends on numerous factors, which are shown in Table 1.

In summary, these factors can be subdivided into the physicochemical conditions that are determined by the environment, the material properties of the polymer to be metabolized and the type of microorganisms present. Only if all listed conditions are measured and controlled, a reliable statement on the degradability of a material and a comparison between degradation processes are possible.

The detailed presentation of all factors goes beyond the scope of one single review. In the environment, however, certain environments with comparable conditions for biodegradation can be identified, which will be presented in the following and considered further in this summary.

Seawater: Oceans cover 71% of the earth’s surface and contain 97% of the world’s water reserves [11]. Seawater is generally characterized by a high salt content of 34–37 ppt. Up to 99% of the salts are ionic compounds with chlorine, sodium, sulphur, magnesium, calcium and potassium [12]. Temperatures range from 30 °C on the surface in summer to −1 °C on the seabed in winter and depend not only on the season but also on the depth and geographical location [13]. Usually, a temperature of 15 °C is assumed for standard seawater. The pH value of seawater is 7.5–8.4, slightly in the alkaline milieu [12]. Only very few fungi can survive in seawater; among the existing bacteria, anaerobic microorganisms dominate, especially in deep layers with low oxygen content, when compared to aerobic microorganisms [14]. In addition to its influence on water temperature, the availability of light also plays an important role in the activity of photosynthetic microorganisms and algae [15].

Fresh water: Fresh water can basically be divided into stagnant and dynamic waters. The environmental conditions are the same as in sea water, but the decisive difference is the low salt content of less than 1 ppt [16]. Temperatures depend on the season, rainfall, location and depth of the water. Lake Constance in Central Europe measures temperatures between 4 and 25 °C [17], whereas temperatures in the African Lake Victoria range only between 24 and 29 °C [18]. The pH value of fresh water is between 6 and 9 [16,19]. Bacteria and fungi are mainly responsible for the biological degradation in fresh water, whereby fungi are predominantly found a few millimeters below the water surface.

Soil: The parameters for biodegradation in soil differ even more than in other environments. The soil texture varies from region to region. A coarse sand with particle sizes up to 2 mm leaves a lot of space for gas diffusion with the environment, while this is strongly impeded in a compact loamy soil with particle sizes < 2 µm. Depending on precipitation and climate, temperatures and pH values vary: the latter can be between 2 and 11. This also influences the population density and activity of microorganisms in the soil [20]. However, aerobic degradation is generally assumed in soil [21]. The conditions in the soil differ according to region and composition to such an extent that even rough guideline values can hardly be given here. The OECD guideline 307 defines a laboratory test for biodegradation in soil. Here, the temperature is selected at 20 ± 2 °C, a duration of 120 days and a pH of 5.5–8 [22].

Composting: The composting environment is often described as a homogeneous ecological niche under aerobic conditions and can be better controlled in terms of composition, pH, humidity and size than biodegradation in soil [20]. Different raw materials can be used as compost material, but mainly green waste and agricultural residues are used. The aim is a relative humidity of 45–60% and a pH value between 6.5 and 8.0 [23]. There is currently no ISO standard or ASTM guideline for home composting conditions. However, the “OK compost Home” certificate is used worldwide. This defines polymers as home compostable if at least 90% (measured after CO_2_ release) is degraded within 12 months at ambient temperatures of 20–30 °C [24].

Industrial composting: Compared to home composting, industrial composting plants operate at elevated temperatures, typically 50–60 °C [25], which increases the activity of thermophilic bacteria and fungi. The period for biological degradation can thus be significantly shortened. In addition, the size and volume of the compost heap differ. Turning and aeration of the compost can further accelerate degradation [26]. DIN EN 14855 specifies a temperature of 58 °C and a maximum duration of 6 months for industrial composting [7]. Due to the similar conditions, industrial composting and composting are considered together in the following.

Sewage sludge: In Europe, about 8 million tons of sludge solids are produced annually, which are obtained in sewage treatment plants by sedimentation. Sewage sludge is an important organic waste because it contains important nutrients and can be used as a fertilizer. Biogas can also be produced from sewage sludge [27]. Due to the organic carbon, phosphorus, nitrogen and other nutrients, sewage sludge enables optimal growth conditions for microorganisms. The microbial population density is, therefore, significantly higher than that of seawater or fresh water [28]. Depending on the composition of the sewage sludge, degradation can be aerobic or anaerobic. Temperatures between 37 and 50 °C are considered as advantageous here [20]. Conditions for the aerobic and anaerobic degradation of polymers in sewage sludge are laid down in the ASTM guideline D5209-92 [29].

Landfill: Analogous to seawater and fresh water, biodegradation takes place in landfills under anaerobic conditions. The anaerobic degradation initially takes place under dry conditions by inoculation with microorganisms from digestion tanks. In order to accelerate the decomposition, moisture and oxygen can be added during and after operation of the plant. The pH value lies between 5.8 and 8.5 [20,30]. Recirculation of the contaminated leachate collected at the bottom of the landfill and returned to circulation to prevent it from entering the soil and ground water can improve the performance of a landfill significantly [31].

**Figure 1:**
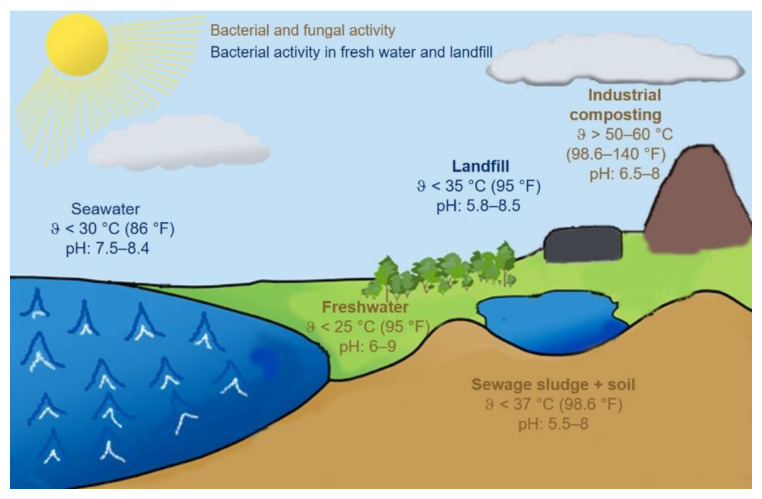
Overview of ambient conditions.

The different environmental conditions with their most important parameters for biological degradation are summarized graphically in Figure 1.

For a direct comparison of corresponding studies, all of the above-mentioned parameters would always have to be investigated and reported. Since this is rarely the case, the statements in this review are to be understood only as a guideline.

## 3. Polymers

**Scheme 1:**
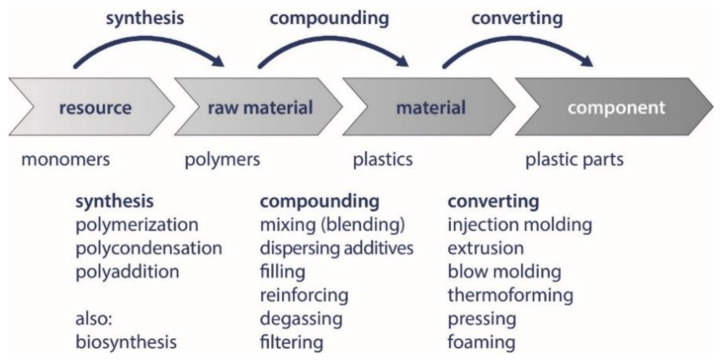
The Value Chain from a Monomer to a Plastic Component [10].

The following is a detailed overview of the degradation of a variety of biodegradable polymers in the environments presented in the previous section. Polymers are often compounded for better processability or supplementary functionalities with additives (see Scheme 1).

One has to keep in mind that these can also influence the biodegradation of matrix polymers. Since PLA is currently the focus of attention due to its ready availability, and the polyhydroxyalkanoates PHB and PHBV due to its ready degradability, these polymers are comprehensively presented. The remaining polymers are evaluated briefly. Due to the active research landscape in the field of biopolymers, this is only a snapshot and does not claim that only these polymers are biodegradable.

### 3.1. Polylactide (PLA)

Polylactide is a biogenic polyester and can be produced by direct polycondensation from the monomer lactic acid, which is obtained on an industrial scale by fermentation from renewable raw materials. Lactic acid is available in two isomeric forms, D- and L-lactic acid. The ratio of the two isomers in the polymer has a major influence on its properties. The most common route to PLA is, however, the ring-opening polymerization of lactide. The glass transition temperature of PLA at 50–80 °C is low compared to most polyesters, which severely limits the application of this, in its pure form, nearly amorphous material. PLA is already available in industrial quantities and in numerous application-specific grades, for example with increased crystallinity, which makes it interesting for the processing industry [25,32,33]. In the following, the biodegradation of PLA in all environments from Section 2 is presented.

In contrast to other polymers that are considered to be directly attacked by microorganisms as described above, the degradation process of PLA is understood to proceed via a sequential mechanism wherein the first step involves a simple chemical hydrolysis to reduce the molecular weight of the PLA. In a second step, the present microorganisms directly metabolize the lactic acid oligomers [34].

Seawater: The low temperatures and low bacteria density in seawater are not suitable for biodegrading PLA. Neither the mass average molar mass *M_w_* nor the Dispersity or the total mass showed a significant change after 10 weeks in real seawater with a water temperature of 25 °C. This result, based on film chips with a thickness of only 0.05 mm, was independent of the crystallinity of the PLA used [35]. In [36], no mass loss of the 0.32 mm thick films was found under simulated conditions, even over a period of one year, which shows the significant influence of the sample geometry. In contrast, a newly developed copolymer of PLA and glycolic acid, poly(lactic-co-glycolic acid) (PLGA), showed a complete mass loss after 270 days. In this case, the formation of lactic acid could be detected by means of high-performance liquid chromatography (HPLC).

Fresh water: Even in fresh water, PLA showed no mass loss under simulated conditions at 25 °C water temperature. Once again, a completely degradable polymer could be synthesized by copolymerization with glycolic acid [36]. The similarity of the seawater and fresh water environments was already mentioned in Section 2, so the similar degradation at identical temperatures is not surprising.

Soil: Based on [37], it becomes clear that the most important factor influencing the biodegradation of PLA is temperature. Under otherwise identical conditions, the biodegradation of film strips with a thickness of 0.2 mm at 25, 37, 45 and 50 °C was compared. Even after one year at 25 and 37 °C, respectively, no loss of mass was detectable; but at 37 °C, a decrease in the molar mass from 160 to almost 70 kDa was measured. However, a mass loss of more than 40% and a significant reduction in the molar mass could be detected after 8 weeks if the temperature was increased to 45 °C. An increase in the temperature to 50 °C reduces this time to only 5 weeks. In [38], the degradation was evaluated on the basis of the tensile strength of the films, which decreased from 53 to 24 MPa over 11 months, indicating embrittlement due to ageing processes. However, neither the molar mass nor the total mass were considered here. Additionally, [39] makes no statements about the total mass loss, showing a decrease in the mean molar mass of about 30% after 12 weeks at a soil temperature of 27 °C.

(Industrial) composting: The high bacterial activity in compost has a positive effect on the biological degradation of PLA. At 37 °C, a total mass loss of 22% could be measured after one year by halving the molar mass. Even at 25 °C, the molar mass had already decreased significantly. By increasing the temperature to 45 (9 weeks) or 50 °C (6 weeks), the loss in mass increased to almost 60% [37]. A decrease in the molar mass from 0.3 mm thick foils to only 7 kDa (M_w,0_ = 105 kDa) was proven in [40]; the temperature was 59 °C here. At 45–70 °C, it took only 17 days to reduce the molar mass from 150 to 4.5 kDa [41].

Sewage sludge: Another method for evaluating the degradation is the calculation of the biological degradation via CO_2_ release. In [42], PLA powder with a particle size of 120–250 µm is investigated in anaerobic sewage sludge. At a temperature of 37 °C, a biological degradation of 43% was detected after 277 days. The same authors performed the experiment in [43] at 55 °C, which increased biodegradation to 75% in only 75 days. In [41], the biodegradation of PLA films 0.3 mm thick in an effluent treatment at 25–32 °C was investigated, and only a decrease in molar mass from 150 to 50 kDa within 15 months was found. Here, the authors themselves recommend that a temperature above the glass transition temperature should be selected for biological degradation of PLA.

Landfill: The degradability test in a real landfill environment showed a decrease in the molar mass of PLA films to 17 kDa. The observed color change, from clear to white-opaque, suggests an embrittlement with an associated increase in crystallinity. Fragmentation of the foils was observed after 11 months, but these fragments were still detectable after 15 months [41].

### 3.2. Polyhydroxybutyrate (PHB)

Due to their ready biodegradability, polyhydroxyalkanoates (PHA) and their most important representative, PHB, have attracted increasing attention in recent years. In contrast to PLA, PHA can be fermented directly in bacteria, which enrich the polymer intracellularly as a storage material [44]. PHB is often compared with polypropylene (PP) in terms of its properties [32], but it is much stiffer and more brittle without prior modification. Compared to other biopolymers, PHB has good barrier properties against water and oxygen and is, therefore, of particular interest for food packaging [45].

Seawater: PHB was proven to be one of the few plastics biodegradable in seawater. In a simulated seawater environment at 25 °C, a total weight loss of 7% was detected after one year using films 0.32 mm thick [36]. It is noticeable that no change in molar mass was detected. In the literature, however, we observe a large variation among the degradation rates of PHB. In [46], a total mass loss of 42% (films 0.1 mm thick) or 38% (granules 5 mm thick) was measured after only 160 days, even if the temperature of 29 °C is very similar to the previous source. The deviation to [35] is even more obvious, where a weight loss of 60 % was already recorded after 35 days. What they all have in common, however, is the observation that molar mass and Dispersity do not change, or change only slightly, so that the degradation of the polymer chains is uniform and does not depend on the chain length.

Fresh water: As seen before in the case of PLA, the degradation of PHB in fresh water does not differ significantly from that in seawater. Bagheri et al. [36] demonstrated a mass loss of 8.5% after one year under simulated conditions analogous to the study in seawater. [47] confirms the biodegradability in fresh water, but a total mass loss of 7% was measured after 180 days, and 35% after 358 days. However, further information on the material used, its geometry or on the prevailing environmental conditions would be desirable.

Soil: The very detailed work of Mergaert et al. [48] investigated the degradation of PHB in various soils with different pH values at different temperatures and humidity over a period of 200 days. In addition, injection-molded tensile test specimens with a thickness of 2 mm were used instead of the thin foils frequently used. The result was a strongly temperature-dependent degradation with a mass loss of 0.05%/day at 15 °C, 0.12%/day at 28 °C and 0.45%/day at 40 °C. The latter strong increase is attributed in particular to the increased fungal activity at temperatures of around 40 °C. In addition, more than 200 different microorganisms were detected. Boyandin et al. [49] also confirmed the biodegradation of PHB in a tropical soil. They observed a loss in mass of 98% after one year in the case of foils, and still 15% in the case of granules (thickness: 5 mm).

(Industrial) composting: Despite increased bacterial activity in compost, in [50], a total mass loss of only 6% was observed on a tensile test specimen made of PHB. The temperature of 6–32 °C is far below the optimum of numerous bacterial cultures. A period of 150 days was considered. At a temperature of 55 °C, Tabasi et al. observed nearly 80% biodegradation, measured as evolution of CO_2_, after 30 days [51]. Complete visible disintegration after only 28 days at 58 °C has also been reported [52].

Sewage sludge: The high bacteria density in sewage sludge is advantageous for biological degradation. Accordingly, at 35 °C, a complete mass loss is detected after 12 weeks for slabs with a thickness of 0.5 mm and after 19 weeks for a slab thickness of 3.5 mm [53]. Biological degradation by CO_2_ release was also investigated here, but the variation of the time periods between the individual experiments only allows the conclusion that a positive detection was possible. However, [54] observed a mass loss of approximately 30% at 30 °C in sewage sludge after 13 days.

Landfill: The latter work also investigated the degradation of PHB in landfill leachate and showed, after 13 days at 30 °C, a mass loss of about 10% [54]. At room temperature, PHB powder is buried in a landfill with a mass loss of more than 90% after 4 weeks [55]. Degradation experiments were also carried out with three specific bacterial strains, which had already completely degraded the PHB powder after 5 or 7 days.

### 3.3. Polyhydroxybutyrate-co-valerate (PHBV)

In addition to the short methyl side chains of the PHB, the copolymer PHBV contains ethyl side chains due to its valerate content. Due to the resulting steric hindrance between the chains, crystallinity and melting temperature decrease with increasing valerate content. The stiffness also decreases with increasing ductility, which has a positive influence on the processability of the polymer. At the same time, the valerate content has a major influence towards an increased degradability of the polymer.

Seawater: Like PHB, PHBV is biodegradable in seawater. At a temperature of 29 °C, a total mass loss of 46% of films 0.1 mm thick, and 13% of granules 5 mm thick over a period of 160 days was observed [46]. The PHBV investigated here has a valerate content of 11 mol%. Matavulj et al. even observed a complete mass loss after 350 days without providing information on the polymer composition or the applied environmental conditions [56].

Fresh water: Studies in Lake Lugano in Switzerland have shown that PHBV with 21 mol% HV is completely degraded within 254 days, even at a temperature of only 6 °C [57]. In [48], the valerate content was also taken into account as an influencing parameter. A PHBV with 10 mol% HV showed a total mass loss of 77% after 358 days; with a 20 mol% HV, a complete mass loss was observed in the same period and under the same conditions. The influence of the geometry becomes clear on the basis of [56]: here, strips were cut from a bottle (30 × 10 × 1 mm). At low temperatures, a mass loss of only 29% was measured after 350 days.

Soil: PHBV is also readily biodegradable in soil. Over a period of 200 days, tensile test specimens made of PHBV with 10 mol% HV showed an average mass loss of 0.09%/day, 0.14%/day and 0.64%/day, at average temperatures of 15, 28 and 40 °C, respectively [48]. A direct comparison with the homopolymer PHB is possible, which proves the better degradability of PHBV. The comparison of film discs with a thickness of 1 mm and granules with a thickness of 5 mm in [49] showed that, after 365 days, 61 % of the film is degraded, whereas only 35 % of the granules is degraded. A complete weight loss of strips from bottles was achieved after 525 days [58].

(Industrial) composting: Comparatively low temperatures are sufficient for the decomposition of PHBV in compost. Strips of PHBV with a thickness of 1 mm showed a total mass loss of 100% after 70 days at 20–24 °C [56]. Tensile test specimens with a thickness of 2 mm achieved a total mass loss of 4% (10 mol% HV) or 30% (20 mol% HV) after 150 days.

Sewage sludge: Briese et al. confirm a complete mass loss of PHBV bottle pieces with 15% HV content after 12 weeks and also show the influence of the pH value on biodegradation [59]. At a pH value below 6, or above 8, the degradation drops to below 10%. In [60], a mass loss of 89% after 100 days was measured in sewage sludge at 35 °C using plastic films made of PHBV. In addition, the carbon content in the gas phase of the hermetically sealed sample increased to 50%.

Landfill: In a simulated landfill environment with leachate, in [46] a total mass loss of 60% could be determined after 350 days. The temperature was 35 °C. However, at a temperature of 20–24 °C in a real landfill, strips 1 mm thick were completely degraded after 280 days [56].

### 3.4. Further Biopolymers

In addition to the three polymers already presented, there are numerous other polymer groups that are biodegradable. In the following, the best-known of these will be presented briefly, and their degradation behavior will be discussed.

#### 3.4.1. Biodegradable Polymers from Petrochemical Sources

Biopolymers that are not produced on the basis of renewable raw materials can also show biodegradation. These polymers include polycaprolactone (PCL), which is obtained by ring opening polymerization of ε-caprolactone. PCL degrades completely in seawater in just a few weeks [35,61,62]. The conditions in anaerobic sewage sludge are also suitable [61], but the slower degradability of 3 mm thick tensile test specimens is again evident, where less than 5% mass loss is detected after 120 days [63]. In direct comparison to this, PCL degrades in soil, but only very slowly [63], which can be explained by a low population density of microorganisms. In compost, complete degradation can be observed within a few weeks [61,63,64].

Polybutylene succinate (PBS) is currently still produced from crude oil, but will in future also be available in a (partially) biobased form. It is degradable in soil, but after 180 days only 11% [65], or 28% in the case of films [66], is degraded. In [67], the discrepancy of the evaluation methods is illustrated: a mass loss of 9% is measured after 180 days, but the determination of the biological degradation via released CO_2_ results in a degradation of 65%. Degradation is also detected in composting environments [64,68,69].

Polybutylene adipate terephthalate (PBAT) is an aliphatic-aromatic, linearly structured copolyester. PBAT degrades slowly in the soil: in [70] a total mass loss of 22% of films 1 mm thick over a period of 168 days is shown. Further information on soil degradation can be found in [71] and [72]. However, recent research clearly shows that PBAT does not degrade in marine and fresh water [36].

Polyethylene glycol (PEG) is an aliphatic polyether. In the long term, production based on renewable raw materials is expected. In the case of PEG, the influence of molar mass on biological degradation was investigated in detail. Up to a molar mass of 10 kDa, granules of PEG are completely degradable in a short time, even in seawater. From 26 kDa upwards, no degradation can be detected at all. In fresh water, molar masses up to approximately 60 kDa are completely degradable [73]. Further studies deal with the chemical analysis of degradation products [74] or with the microorganisms involved in degradation [75,76].

#### 3.4.2. Cellulose-Based Biopolymers

Cellulose is the most abundant organic material in the environment [32] and, due to its ready biodegradability in all environmental conditions, it is often used as a positive reference material in biodegradability tests, for example in ASTM D5209-92 [29]. In addition to bacteria and fungi, unicellular protozoa active in water can also metabolize cellulose [77]. There are studies on the positive degradation in seawater [14], in sediments [78] as well as data on the resulting degradation products, for which, in addition to hydrogen and carbon dioxide lactate or acetate also count [79]. The period for the degradation is comparatively short: after only one month in anaerobic sewage sludge and at low temperatures of 10–17 °C, a total mass loss of 40% was proven [80].

Cellophane is produced from cellulose by chemical processing and is mainly used in the form of films, for example in food packaging [44]. Cellophane is also readily biodegradable, and is used in [79] as a positive reference material in a landfill environment, having similar degradation rates compared to PHBV. Further work shows degradation in an aqueous solution [81] or sewage sludge [60]; the latter also shows slower degradation due to the use of coated cellophane. All of the defined environmental conditions are suitable for degradation [44].

The esterification of cellulose allows the production of various cellulose acetates which are predominantly thermoplastic. The most important cellulose esters are cellulose acetate (CA), cellulose propionate (CP) and cellulose butyrate (CP) [82]. The biological degradation of these esters depends on the respective acid residue, the number of ester groups and their distribution, and the degree of polymerization. In general, the degradability decreases strongly with increasing ester groups. In a laboratory test in sewage sludge at 25 °C, cellulose acetate was shown to biodegrade by 10% after 22 days, compared to 70% for cellulose, on the basis of CO_2_ development [83]. Other studies, however, report complete degradation within 10–12 days in landfill [84] and in sewage sludge at 30 C [85].

Cotton consists of 95–97% cellulose [86] and plays an important role in textile technology. Cellulose in cotton cloth has a high degree of crystallinity, of up to 73%, which makes biological degradation more difficult. In amorphous regions, the molecular chains are less strongly bound to each other and thus more easily accessible for an enzymatic attack [87]. According to [88], a cotton T-shirt takes 25 months to degrade completely in seawater.

Pulp is mainly obtained by the chemical decomposition of wood or straw, whereby lignin and hemicellulose are removed. Depending on the cellulose content of different types of wood, a different proportion of pulp is obtained from the wood mass [82]. Even under adverse conditions (nutrient-poor seawater at 1–14 °C), a bleached, lignin-free sulfite pulp can be biologically degraded, in this case; however, after 120 days, only a total loss of 10% is detectable [80]. Correspondingly, pulp decomposes faster in fresh water and sewage sludge, even in the case of industrial wastewater with high lignin content. In particular, the enzymes of various fungi are able to fully utilize pulp [89].

#### 3.4.3. Starch-Based Biopolymers

Starch can be obtained from corn, potatoes or wheat and, thus, from renewable raw materials. Processing consists of numerous steps until the pure starch is isolated. Native starch has many polar groups, which ensures the formation of hydrogen bonds and, thus, high water absorption. It is assumed that starch is completely biodegradable in all environmental conditions, but the authors are only aware of studies on the degradation of native starch in compost. They show that starch films degrade completely within a short time (30–84 days), but cellulose, as a reference material, requires only 10 days under the same conditions [90,91,92]. It should be noted, however, that these investigations carried out composting at an elevated temperature of approximately 60 °C.

By processing native starch with natural plasticizers and plasticizers such as glycerine or urea, a thermoplastic material can be produced. This thermoplastic starch (TPS) is completely amorphous and is usually used in combination with other polymeric materials [82]. Studies on films with a starch content of 85% show slow degradation in seawater [93,94], but the period of about half a year for seawater degradation is comparatively short. The degradation in soil takes place much faster; in [95] and [96], a total mass loss of 100% and 72.6%, respectively, was measured after 30 days at temperatures of 30 and 25 °C. It is interesting to note that, under industrial composting conditions, only a total mass loss of 45% was detected after 49 days [90]. In another study, only 40% was detected after 70 days [97].

Since the processing of pure TPS represents a great challenge, it is often blended with other polymers in order to increase the range of application of the material. Studies on blends of TPS with PCL are particularly frequent, which significantly improves moisture resistance. The biodegradability of these blends is very good; in seawater, a complete weight loss is achieved after only 5 weeks [61,62]. The example of sewage sludge shows again the great influence of the sample geometry: in [63], a total mass loss of 29% after 111 days on tensile test specimens was shown, whereas [61] reports a complete mass loss in sewage sludge after only 4 weeks. Further investigations confirm the degradation in soil [63,98] and in composting environments [63,91,99].

#### 3.4.4. Protein-Based Polymers

Proteins are polymers made up of amino acids and are, therefore, like cellulose and starch, of natural origin. As the main protein in milk, casein is one of the most frequently-represented protein-based polymers. In an isotonic saline solution, casein already shows a total mass loss of 45% after 30 days [100], but the release of ammonia during degradation affects many microorganisms and inhibits their activity [101,102]. In contrast, a low salt content and a slightly alkaline environment cause a significant loss of mass within a few days [103].

Gluten is predominantly the water-soluble protein component in wheat flour and is often used as a binding agent. Like thermoplastic starch, it is dependent on the use of plasticizers for processability. Gluten degrades completely in soil in 30–50 days [104,105,106]; for degradation in composting environments, values between 15% total mass loss in 70 days [107] and 100 % biological degradation in 12 days can be found [108]. In combination with sewage sludge, biological degradation of 85% was detected in 36 days [104]. The results in [109] are interesting, where the mass loss of the two main components of gluten, gliadin and glutenin is determined to be 100% and 30%, respectively, after only one day. The medium used here is distilled water, which suggests the assumption that, instead of actual degradation processes, only water solubility is detected here.

Wool is also a natural biopolymer and differs from casein and gluten mainly in a higher sulphur content due to the amino acid cysteine. The biological degradation of wool, therefore, releases methane and carbon dioxide as well as highly toxic hydrogen sulfide [110]. In seawater, damage to the surface of the fibers is already visible after 21 days, and in [111] a perforated surface after 3 months is reported. Currently, more than 299 different fungal strains are known whose enzymes can degrade wool [112]; a rapid degradation in soil is detectable in a few months [113,114].

#### 3.4.5. CO_2_-Based Biopolymers

The conversion of CO_2_ into polymeric materials represents a possible recycling option for the greenhouse gas. The copolymerization of carbon dioxide with propylene oxide produces polypropylene carbonate (PPC). High temperatures are advantageous for the biological degradation of PPC. In garden soil, a total mass loss of only 3.2% was observed after 6 months [115]. At 40 °C in a composting environment, a mass loss of 8%, accompanied by a strong increase in the Dispersity, was measured [116]. Under the conditions in an industrial composting plant (60 °C), a complete total mass loss can be detected after only 3 months [117].

For the polymerization of polyethylene carbonate (PEC), carbon dioxide is copolymerized with ethylene oxide instead of propylene oxide. PEC is mainly used in the pharmaceutical industry as a coating agent or in medical technology for biodegradable stents. The literature, therefore, only provides studies on biological degradation in vivo. Implantation in rats resulted in a total mass loss of 50% after 2 weeks for foils [118]. In [119], a complete mass loss of granules is achieved after 14 days. The degradation behavior of PEC is also largely dependent on its molar mass, the carbonate content of the polymer chain and the catalyst used [120].

#### 3.4.6. Other Biopolymers

There are a multitude of biodegradable polymers based on natural sources. Examples are fibers of (spider) silk that are biodegradable in aqueous solutions [121,122] and the scaffold substance chitin that biodegrades slowly in soil [123,124,125].Chitosan is very similar to chitin and needs several months to biodegrade in soil [123,125,126]. Lignin is the main component of the plant cell wall, and biodegrades very slowly, even at elevated temperatures [127,128,129,130], which is also the case for mechanical pulp with a high content of lignin [128,129].

## 4. Key Challenges for Biodegradation Tests

At the beginning of this review, the large number of parameters which influence the biological degradation of polymers was discussed. The results from various experimental studies presented in the previous section show the difficulty in comparing these studies.

In order to create analogies, clearly defined conditions are necessary. Due to the complexity and the high regional dependence, it is generally almost impossible to carry out reproducible investigations in the environment. In the laboratory, the necessary parameters are easier to record and control, so that standardized laboratory tests are advantageous for a comparison of the results. It is also indispensable to consider a defined period of time, as it is usually not known whether the degradation is linear or if the degradation rate changes over time. There are p. ex. studies which propose a three-step degradation process for PHB, whereas only two stages are distinguishable for PCL [131].

Numerous studies use the parameter of total mass loss to assess biodegradability. The mass loss, however, is not only caused by degradation but is also influenced by other parameters, such as currents, UV light and thermal or mechanical stress. According to ASTM D6400 [6], ISO 14855 [7] and EN 13432:2000 [5], biological degradation is defined by the amount of CO_2_ released. Further evaluation methods are: the measurement of the increase in surface, the microbial growth, the change in molar mass, chemical element analysis or a comparison of the mechanical characteristic values.

Another aspect that has seldom received enough attention in previous studies is the sample material used. In addition to the chemical composition or the indication of a clear type designation, the geometric shape of the sample also has a major influence on the duration of degradation. A powder offers a high surface-to-volume ratio, which means that more microorganisms can settle on the polymer surface than in a compact tensile test specimen. Even films differ in thickness and surface roughness and thus offer different surface qualities for the colonization by microorganisms.

## 5. Conclusions and Outlook

Plastics in the environment have long since become a topic of worldwide interest. Plastics drift in the world’s oceans, pollute water and soil and have now been detected in remote areas such as the Arctic. This development has also left its mark on humans, even though the health consequences of this exposure to plastic particles cannot be foreseen. Biodegradable plastics can make a positive contribution, at least in applications that inevitably end up in the environment.

The biological degradation depends on numerous environmental conditions; these are in addition to the prevailing physico-chemical conditions and the activity of existing microorganisms, especially the material properties of the considered plastic component. However, the most important environments can be summarized. The most favorable degradation conditions are found in composting environments. In home compost, as well as in industrial composting plants, there is a large microbial diversity with high activity, especially with a good oxygen supply. In the latter case, there are also increased temperatures, which further promote the activity of the microorganisms. Numerous microorganisms are still present in the soil and in sewage sludge, but the temperatures are partly subject to large regional fluctuations. Aqueous environments (fresh and seawater) show the lowest biological activity, because the water provides a strong dilution. In landfills, biological degradation takes place at a slower rate, and under the exclusion of oxygen, and is strongly dependent on the mode of operation.

**Figure 2:**
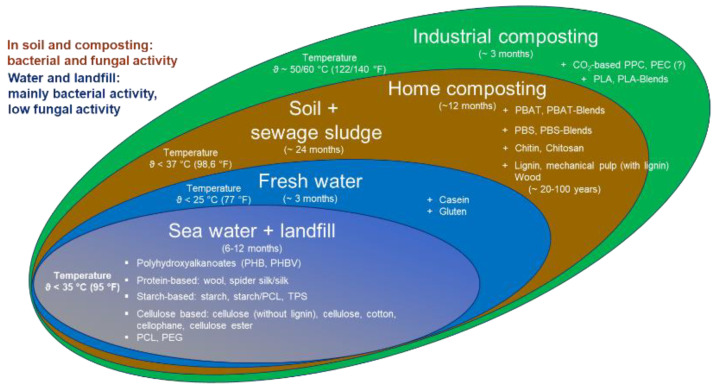
Biological degradation of different polymers in different environments.

The aim of this review was to compare the biodegradability of biopolymers in various environments. The results are summarized in Figure 2.

However, despite extensive literature on the subject, it is almost impossible to make generally valid statements. As mentioned above, the decisive factors for biodegradation are manifold and rarely documented in detail. In most cases, the available results differ or even contradict each other, making it impossible to specify a defined degradation period. In order to be able to make reliable statements, it is essential to clearly define specifications with regard to physico-chemical conditions, performance, test specimen geometry, etc. Possible standardizations are offered by the guidelines presented (see Box A2 in the Appendix A). In particular, however, the measured target must be standardized. Due to the aforementioned influencing factors on the total mass, only the determination of the biological degradation via the CO_2_ release allows a direct conclusion on the actual metabolism of a polymer.

## Figures and Tables

**Table 1:** Major influential factors for biological degradation.

Physicochemical Conditions	Material Properties	Enzymatic Effects
Moisture/water content pH value Temperature Availability of oxygen Availability of nutrients Redox potential	Molar mass Polymer composition Steric configuration Size, shape and surface area Melting and glass transition temperature Polymer crystallinity Porosity Material thickness Additives Fillers	Microbial activity Microbial diversity Microbial population density

## Appendix A. Explanatory Boxes

Box A1 The Difference between Biodegradability and Compostability. Biodegradability: Biological process of organic matter which is completely transformed by microorganisms (bacteria and fungi) into water, CO_2_/methane, energy and new biomass → material property. Compostability: certified biodegradable material that is converted into compost under controlled conditions (e.g., composting plants) within a certain time frame → product property.

Box A2 Existing Standards and Certifications for Biodegradation of Polymers in Europe and Worldwide. ISO 14855: Method for determining the aerobic biodegradability of plastics under controlled composting conditions by measuring the amount of carbon dioxide developed and the degree of decomposition of the plastic at the end of the test. A variant of the process is also specified, in which, instead of mature compost, a mineral bed (vermiculite) is used, which is inoculated with thermophilic microorganisms [7]. ASTM D6400: Establishment of standards for the identification of plastic products and materials composted in aerobic composting plants. This includes biodegradation at a rate comparable to known compostable materials [6]. The standard is the regulatory framework for the United States and is comparable to EN 13432. EN 13432: Must be fulfilled for a plastic product to be compostable on the European market. The standard contains several tests and defines the criteria for passing or failing: the decomposition of the finished product within 12 weeks, and the biodegradation of the polymeric components within 180 days, as well as a study of plant toxicity and heavy metal content [5]. OECD 301A: Degree of biodegradation through the change in dissolved organic carbon over a period of 28 days. This involves testing for slight or complete biodegradation [132]. OK compost Industrial: Products and packaging carrying the OK compost logo are guaranteed to be biodegradable in an industrial composting plant (Industrial) [133] or in a garden compost (Home) [24]. The reference for this certification is the European standard EN 13432:2000. OK biodegradable Marine/Water: This international certification guarantees the biological degradation in the sea [134] or in natural fresh water [135]. OK biodegradable Soil: Products with this logo are fully biodegradable in soil and have no negative impact on the environment [136].
